# Inheritance and advancement in biochemistry: the pioneering research of Szu-Chih Liu

**DOI:** 10.1093/procel/pwaf030

**Published:** 2025-05-26

**Authors:** Quanxiu Li, Cheng Zhen

**Affiliations:** School of Health Humanities, Peking University, Beijing 100191, China; School of Health Humanities, Peking University, Beijing 100191, China; Center for the History of Medicine, Peking University, Beijing 100191, China

Szu-Chih Liu (刘思职, 1904–1983) (Fig. 1) was a pivotal figure in the history of biochemistry in the 20th century China, and the year 2024 commemorates the 120th anniversary of his birth. At the commencement of his biochemistry journey for an extended period of more than five decades, he worked as a competent colleague of Professor Hsien Wu in the Department of Biochemistry at Peking Union Medical College (PUMC), elucidating the structural mechanism for protein denaturation and quantitatively examining antigen-antibody equilibrium in immunochemistry. He subsequently established the Department of Biochemistry at the College of Medicine, National Peking University, which was later reorganized into Beijing Medical College (now Peking University Health Science Center), where he led to explore a plethora of intriguing biochemical problems, as well as nurtured generations of talents. He initiated pioneering efforts to localize biochemistry in China through sustained efforts on unifying biochemistry terminology and developing biochemistry textbooks in the Chinese language. He was elected as an academician of the Division of Biology in the Chinese Academy of Sciences in 1957.

**Figure 1.**
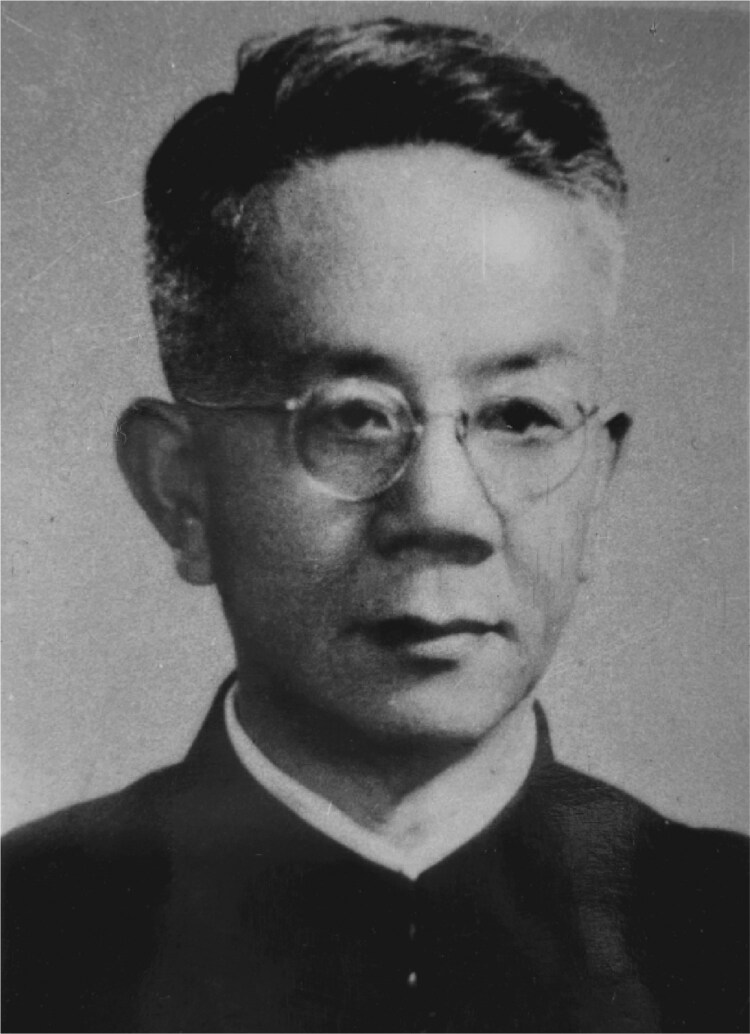
Professor Szu-Chih Liu (1904–1983).

Professor Szu-Chih Liu possessed broad interests in extensive research areas encompassing protein denaturation, immunochemistry, ultrasonic radiation, ammonium metabolism, and other topics. From PUMC to Beijing Medical College, Liu consistently emphasized the research conducted should be the most cutting-edge worldwide.

## Elucidating the structural mechanism of protein denaturation

The first research focus of Szu-Chih Liu was to elucidate the molecular mechanism of protein denaturation. The essence of protein denaturation had long remained enigmatic, and had been previously supposed to be a process of either hydrolysis or anhydride formation. In PUMC, Hsien Wu, Szu-Chih Liu, and colleagues managed to resolve the mystery of denaturation and conducted a myriad of systematic biochemical analyses since 1924 (Zhu et al., 2004, 2019). In the 13th International Physiological Congress held at Harvard Medical School in Boston in August, 1929, Hsien Wu unveiled the groundbreaking proposal that the protein molecule “is not to be regarded as a long straight chain but rather as a compact structure” and the chain “may be conceived to fold repeatedly at short intervals forming a three-dimensional network…” (Du and Gu, 2019; Wu, 1929). This was the earliest accurate three-dimensional depiction of protein structures in solution (Rall, 2016). He also realized that the denaturation process was purely a change in three-dimensional structure involving protein unfolding rather than a protein chemical reaction as previously conceived:

Denaturation is the breaking up of these labile linkages. Instead of being compact the protein molecule now becomes a “diffuse” structure. The surface is altered and the interior of the molecule is exposed. This explains the decrease in solubility, increase in acid and base binding power, and the change in immunological specificity which are known to accompany denaturation (Wu, 1929).

In 1931, they comprehensively proposed the theory of protein denaturation for the first time that a soluble protein molecule was “not a flexible open chain of polypeptide but has a compact structure” held together by the “force of attraction between the polar groups in a single molecule,” and “the compact and orderly structure” was disorganized in the process of denaturation (Wu, 1931, Edsall 1995).

A widely debated controversy existed in the late 1920s over whether the protein solution pH would change or remain unaltered during denaturation. In 1931, Hsien Wu, Szu-Chih Liu, and others illustrated pH of protein solutions underwent mild changes during denaturation, indicating that the protein’s intrinsic capacity for binding H^+^ or OH^−^ was altered accordingly (Wu et al., 1931). They quantitatively characterized the acid titration curves of both native albumin protein and heat-denatured albumin protein, and the titration curve of heat-denatured protein evidently shifted towards the alkaline side compared to that of the native protein.

The urea denaturation approach precluded common complications of decomposition and coagulation that occurred in thermal denaturation. Three albumin solutions added with urea all exhibited an obvious rise in pH as time elapsed. Additionally, the pH of controls of protein or urea alone showed no sign of change.

They further measured the pH change of protein denaturation by alcohol. Solution pH experienced an increase as denaturation proceeded, refuting the previous view of no change in pH during alcohol-induced denaturation (Booth, 1930).

The aforementioned pH changes in denaturation collectively showed the change of acid or base binding power of protein in the course of denaturation. It was implied that substantial three-dimensional conformational changes occurred during the protein denaturation, thus the protein surface landscape of amino acid side groups for protonation was altered. Such observation corroborated Hsien Wu’s theory of protein denaturation.

Liu also demonstrated that urea-induced albumin denaturation was accompanied by an increase in solution viscosity. Since compact, well-folded proteins generally exhibited lower viscosity, these results also suggested that urea denaturation led to an extended or unfolded protein structure (Liu, 1933).

## Innovative endeavors in immunochemistry

The second field of study of Szu-Chih Liu was immunochemistry. His excellent expertise in analytical and physical chemistry greatly facilitated his exploration in immunochemistry, where chemical equilibrium principles and quantitative analytical approaches were elegantly applied in immunochemistry studies. He launched his seminal work in immunochemistry in the 1930s and maintained a burgeoning array of research interests all through his academic career.

A practical method for fractionation of serum proteins using methanol at low temperature, maintaining proteins in native states, was developed by Liu and Wu (Liu and Wu, 1934a). Applying this method, Liu and coworkers investigated the effects of bacterial immunization on serum protein distribution in rabbits and horses. The protein fraction containing antibodies showed a substantial increase following immunization, while it decreased rapidly upon discontinuation of immunization (Liu et al., 1937a). They also explored the effect of lipid removal on the solubility and precipitability of proteins (Liu and Wu, 1937a, 1937b). The work of others seemed to indicate the absence of the basic globulin in serum. Liu, Wu, and colleagues, however, showed that basic globulin was present in both normal serum and immune serum of horse (Liu et al., 1937b). The majority of antibodies were identified to be in the basic globulin fraction of immune horse serum, and antibody in horse sera samples increased after immunization against pneumococcus.

In the late 1930s, they investigated the possibility and mechanism of antibody recovery from antigen–antibody immune precipitate (Liu and Wu, 1939c). The agreeable pH range for antigen–antibody combination was 5 to 9. In solutions of acid pH < 5 or alkali pH > 9, antigen–antibody combination was incomplete. If immune precipitate in neutral was treated with dilute acid or alkali, the shift of antigen–antibody immunochemical equilibrium would drive the partial release of free antibodies from the immune precipitate, illustrated as follows (Liu and Wu, 1938b).


$$\begin{array}{l} B_{m}G_{n}\;\begin{array}{l} \overset{acid\;\;\;or\;\;\;alkaline}{\to }\;\;\underset{\;\;\;neutral\;\;\;\;\;}{\leftarrow }\;\; \end{array}\;B_{m-x}G_{n}\;\;+\;\;xB \end{array}$$

where B was antibody, G was antigen, and B_m_G_n_ indicated the immune precipitate formation in pH neutral solution. Upon the action of acid or alkali, a shift of equilibrium occurred, causing a portion of antibody xB to be released from the immune precipitate. Even if the mild acid or alkali were able to liberate antibodies from the precipitate, too strong the acidic or alkaline conditions might dissolve the precipitate, decreasing antibody recovery. They then sought to identify the optimal pH conditions for the recovery of horse and rabbit antibodies from immune precipitates of pneumococcus. In 1938, the recovery of anti-egg-albumin antibodies was performed (Liu and Wu, 1938a). The antibody was also recovered from the immune precipitate against another bacterium, with the optimal pH for antibody recovery identified (Liu and Wu, 1942). The antibody recovery from the immune precipitate constituted an important approach for antibody isolation and preparation.

Furthermore, they explored the possibility and mechanism for antigen recovery from antigen–antibody immune precipitate (Liu and Wu, 1940c). As previously described, the treatment with moderate acid or alkali liberated a portion of antibody from immune precipitate, resulting in B_m–x_G_n_, which contained a higher level of antigen than neutral precipitate. When neutralized, it should release a part of antigen as follows (Liu and Wu, 1940b).


$$\begin{array}{l} B_{m-x}G_{n}\;\overset{neutral}{\to }\;\;B_{m-x}G_{n-\frac{n}{m}x}\;\;+\;\;\frac{n}{m}xG \end{array}$$

Notably, in neutral environment, B_m–x_G_n–nx/m_ and B_m_G_n_ shared equivalent antigen-antibody stoichiometry: (m–x)/(n–nx/m) = m/n. Antigen was successfully recovered from pneumococcus horse immune precipitate, further substantiating the theoretical hypothesis of antigen–antibody immunochemical equilibrium, as well as the acid/alkali-induced shift in equilibrium. Their findings suggested a feasible approach for antigen isolation and preparation.

The quantitative characterization of the antibody–antigen stoichiometry in immune precipitate further underpinned the antigen–antibody immunochemical equilibrium hypothesis (Liu and Wu, 1940b). B_m_G_n_ and xB of egg albumin immune precipitate were identified to be B_13_G_4_ and 9B, agreed fairly well with the previous calculation of an immunologist (Heidelberger, 1938). They also quantitatively investigated hemoglobin-anti-hemoglobin precipitate, in which B_m_G_n_ and xB were B_13_G_4_ and 5B (Liu and Wu, 1940b).

Liu and Wu quantitatively studied the effect of one antigen on the immune response of another antigen during simultaneous immunization with both antigens. In dual immunization, a second antigen reduced the immune response of the first antigen. However, the overall antibody production following immunization with both antigens was higher than that observed with a single antigen (Liu and Wu, 1939a). The individuality of antibody behavior in serum was elucidated, showing that each antibody reacted only with its corresponding antigen and not with the other antigen during simultaneous immunization (Liu and Wu, 1939b).

The injection of a crystalline globulin prepared from watermelon seeds successfully induced immunization reactions in both rabbit and horse sera (Liu and Wu, 1940a). The quantitative analysis of immune precipitate solubility revealed that immunization with even a pure crystalline protein in mammals could generate distinct antibody species, each of which was capable of forming a dissociable antibody–antigen complex (Liu and Wu, 1941). A brief examination of the presence of protein antigen in urine following antigen injection was conducted (Liu and Wu, 1949). An intriguing observation was made that, in rabbits that failed to respond to egg albumin immunization, an elevated level of serum globulin was detected, suggesting that the production of extra non-specific globulin was also a consequence of immunization (Liu, 1949).

It was putatively discovered in the late 1930s that rabbits, upon immunization with a pure antigen, exemplified by crystalline egg albumin, produced both complete functional antibody and malfunctioned antibody in their sera. The latter was designated low-grade antibody or incomplete antibody. The complete antibody would readily precipitate with its antigen, whereas the low-grade antibody failed to precipitate independently with antigen. Szu-Chih Liu and his student Shih-Chung Wang (王世中) identified the low-grade antibody could also be precipitated only in the presence of both the antigen and the complete antibody (Liu and Wang, 1958). They quantitatively identified the low-grade antibody constituted 14%–30% of total antibody content, and found the low-grade antibody still possessed specificity. They also discussed the possible biosynthetic mechanism of low-grade antibody.

**Figure 2.**
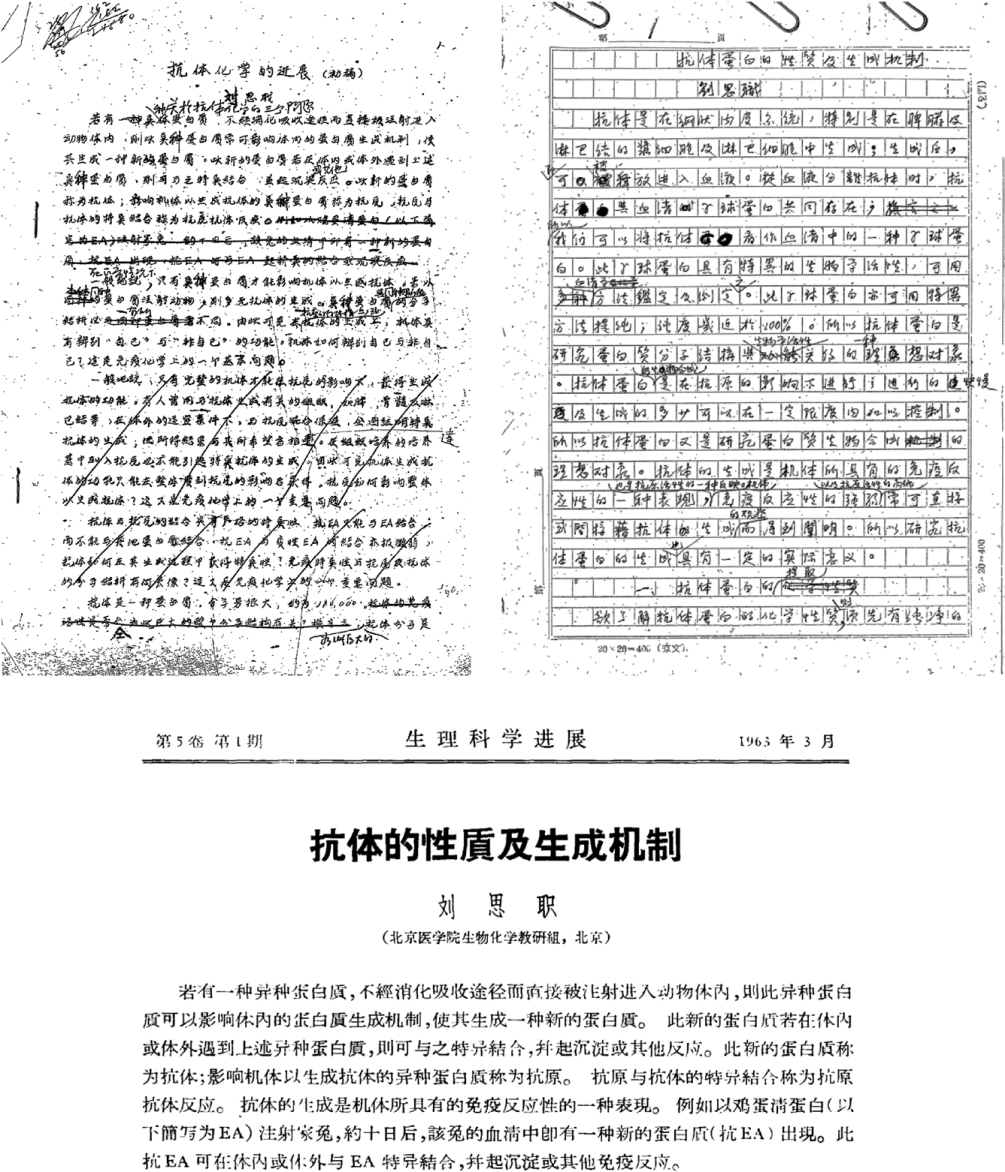
**The evolution of Professor Szu-Chih Liu’s review article manuscript on antibody** (Courtesy of Peking University Health Science Center Archive). (Left) Initial draft entitled “The progress of antibody chemistry”; (Right) Revised manuscript with updated title “The property and production mechanism of antibody proteins”; (Bottom) Final 1963 publication “The property and production mechanism of antibody”.

In 1963, Liu composed a review article that extensively discussed the properties and biosynthesis of antibodies (Liu, 1963) (Fig. 2). With visionary insight, he raised five pivotal issues as the roadmap in the quest of immunochemical research. First, how did antibodies distinguish between “self” and “non-self” molecules? Second, why was the intact immune system required for antibody biosynthesis? Third, what were the differences between common protein synthesis and antigen-induced antibody production? Fourth, how did antibodies acquire specificity during biosynthesis? Fifth, was there an independent reaction center within the antibody molecular structure? Regarding the two prevailing hypotheses of antibody production at that time—the template theory and clonal selection theory—Liu pointed out that although these hypotheses could serve as references for ongoing investigations, their inferences might be completely incorrect due to a lack of experimental evidence (Dong, 2019). He further noted, in the rigorous scrutiny of further research, the erroneous viewpoint would be discarded, and only the valid ones would thus be established. Liu considered DNA as the cornerstone in antibody production and emphasized the antibody biosynthesis followed the central dogma from DNA to mRNA to protein. Given that the central dogma was proposed by Francis Crick in the late 1950s and mRNA was not discovered until 1961, Liu’s assessment of antibody synthesis, which predated the widespread acceptance of the central dogma, highlighted his remarkable foresight (Brenner et al., 1961; Gros et al., 1961).

In a concise communication, Liu discussed the individuality of antibodies, the synergistic or antagonistic effects of different antigens, and other issues pertinent to the simultaneous immunization (Liu, 1965). The diverse opinions from other renowned Chinese immunologists and microbiologists were also fully presented at the end of the communication, including Samuel Zia (谢少文), He Yu (余㵑), and Wengui Chen (陈文贵).

Szu-Chih Liu and his student Zhiwei Dong (董志伟) examined the effect of hormones on antibody metabolism (Dong and Liu, 1966). Two hormones, glucocorticoid cortisone acetate and androgen testosterone propionate, were widely used in clinical practice. It was found in rabbits that both hormones had little impact on the anti-egg-albumin antibody metabolism. In the subsequent test for immunological memory ability to egg albumin, cortisone acetate was shown to suppress the immunological memory, lowering the peak of antibody production, while testosterone propionate exerted no obvious impact.

In the early 1980s, Szu-Chih Liu, with his students Gang Li (李刚) and Guoguang Du (杜国光) compared the metabolic rates and immunogenicity of two different antigens: chicken egg albumin and dog hemoglobin. This turned out to be the final scientific inquiry of Liu’s life (Li et al., 1982, 1984). After a single dose injection of either of the two antigens into rabbits, three rabbits that received albumin showed a notable presence of antibodies, while the four rabbits injected with hemoglobin only showed a very small amount of antibodies. This indicated albumin was a stronger antigen, while hemoglobin was a weaker antigen. In the comparison of antigen intake in different organs or tissues, the liver was found to retain a higher amount of both albumin and hemoglobin antigens than the spleen or lymph nodes, suggesting liver was the major organ for exogenous antigen uptake. Most antigens were cleared from blood within one hour of injection. The albumin antigen exhibited a much higher metabolic rate or decay rate than the hemoglobin antigen across different organs and tissues. It was plausible to suggest that the stronger antigen with higher immunogenicity experienced a faster metabolic rate within organs.

## Exploring the effect of ultrasonic radiation on proteins and bacteria

The third area of investigation of Szu-Chih Liu was the application of ultrasonic radiation during the first half of the 1930s.

It was observed in the late 1920s by others that the egg albumin solution exposed to ultrasonic radiation became turbid, and albumin precipitates, in the shape of fine shreds, were formed (Schmitt et al., 1928). However, it was uncertain whether the solution turbidity was caused by the mechanical vibration of ultrasound itself or by the heating effect accompanied during exposure. Wu and Liu extended their observation with the precaution to cool the solution. Their solution temperature rose no more than two degrees above room temperature excluding the possibility of heat-induced coagulation. Scrupulous observation showed the coagulation of albumin throughout the solution immediately upon ultrasonic waves, and the coagulation was composed of shreds enclosing air bubbles under the microscope. To probe the role of air bubbles in coagulation, they also prepared a gas-free albumin solution with no contact with air. It was found that the gas-free albumin solution displayed no sign of coagulation, suggesting air bubbles were essential and indispensible in ultrasound-induced albumin coagulation (Wu and Liu, 1931).

Other researchers reported the change of color in the solution with a chemical indicator after ultrasonic radiation and suggested ultrasound exposure gave rise to protons, thereby altering pH (Schmitt et al., 1929). Liu and Wu examined and repeated their experiments, concluding that the color change in the chemical indicator was resulted from the oxidative destruction of the indicator molecules provoked by ultrasound, rather than by the production of protons (Liu and Wu, 1932a). Twelve common chemical indicators were applied in the following investigation. The unbuffered solution of each indicator, saturated with air or oxygen, adjusted to show an alkaline color, was exposed to ultrasonic radiation. Ten minutes of radiation exposure turned the color of all indicator solutions lighter than the unexposed control. Some of them even faded to colorless, though none exhibited acid color. The buffered solution of each indicator produced identical results. Nevertheless, when exposed to radiation, the colors of both gas-free solutions and those saturated with hydrogen gas remained unfaded. The solution temperature never exceeded 35°C, ensuring no heat-induced color fading. The above observations elucidated the mechanism of chemical indicator color change during radiation, showing the chemical indicator molecules were permanently destroyed by ultrasound-induced oxidation.

In their continuing investigation into the oxidation mechanism triggered by ultrasonic radiation, they determined radiation directly activated oxygen molecules, with cavitation playing a crucial role. It was suggested that only the oxygen molecules on the surface of bubbles remained active during radiation, and once the radiation halted, the oxidative activity ceased as well (Liu and Wu, 1934b).

In the late 1920s, the destructive effect of ultrasound on bacteria was observed; however, the mechanism behind destruction remained unclear. Liu and his colleague threw some light on the process, suggesting bacteria destruction by the ultrasonic wave was accompanied by bacterial cell lysis and decomposition of bacterial cells. The prolonged ultrasound treatment probably led to protein coagulation following cell lysis (Yen and Liu, 1934).

They further deciphered the mechanism underlying the destructive effect of ultrasound on bacteria. The bacteria suspension was subjected to ultrasonic exposure while being maintained below 20°C, contained within a cooling coil through which cold water continuously circulated to eliminate the possibility of heat-induced bacteria destruction. The bacteria were suspended in various solutions prior to radiation exposure: one saturated with air, one saturated with hydrogen gas, and a gas-free solution. No sign of bacterial destruction was observed in the gas-free suspension. Both the air-saturated and hydrogen-saturated solutions exhibited a reduction in bacteria, though the decrease was more noticeable in the air-saturated solution compared to the hydrogen-saturated one. These findings indicated that gas, whether air or hydrogen gas, was critically involved in bacterial destruction, with the cavitation of dissolved gas being particularly important for the lysis and decomposition of bacteria (Liu and Yen, 1934).

## Delving into the blood ammonium metabolism

The fourth investigative focus of Szu-Chih Liu was blood ammonium metabolism. In the first half of 1960s, Liu led his team to investigate ammonia toxicity, tolerance, metabolism, and clinical interventions.

Abnormally high blood ammonia levels rendered severe toxicity in both humans and animals, which made ammonia metabolism and detoxification a crucial physiological process. The conversion of ammonia into urea in the liver was a key mechanism for ammonia metabolism and detoxification. Another important pathway of ammonia metabolism involves the incorporation of ammonia into glutamate to form glutamine. The liver was the central organ responsible for ammonia metabolism. The removal of liver in animal resulted in severe ammonia toxicity, and human liver diseases such as hepatitis and cirrhosis were often associated with elevated blood ammonia compared to healthy individuals. Szu-Chih Liu and his young colleague Ming Chen (陈明) developed practical methods for the determination of the concentrations of free ammonia, glutamine, and glutamate levels in blood samples from 35 healthy individuals (Chen and Liu, 1964b).

In 1950s, worldwide controversy existed over whether glutamate was effective in the alleviation of blood ammonia toxicity. Since glutamate could combine with ammonia to produce glutamine, it was clinically administered for ammonia detoxification. However, there were huge discrepancies in its clinical efficacy. They measured the levels of free ammonia, glutamine, and glutamate in blood samples from 53 patients with liver diseases, and found the free ammonia in blood was 2–3 times higher than that in healthy individuals (Chen and Liu, 1964a). Although the ammonia level had doubled or even tripled, its concentration still remained lower than that of glutamate in terms of stoichiometric ratio. In other words, glutamate persisted as abundant in patient blood with liver disease, making it reasonable to infer that the administration of glutamate lacked biochemical support and was therefore deemed clinically ineffective.

Blood ammonia tolerance and metabolism were examined in cirrhosis rats by Szu-Chih Liu and his student Shishu Chen (陈诗书) (Chen and Liu, 1964c). The ammonia concentration in blood samples from cirrhotic rats was only around 30% higher than that of normal rats, yet their capacity to tolerate exogenous ammonium chloride was significantly reduced. In normal rats, the administration of ammonia chloride led to a three-fold increase in blood ammonia level within 1 h, with levels returning to normal 2 h after administration. In contrast, when cirrhotic rats were given the same dose of ammonium chloride, their blood ammonia levels rose by more than 20-fold, leading to convulsions, coma, or even death. Moreover, arginine was shown to greatly detoxify ammonium chloride by enhancing urea biosynthesis. When arginine chloride and ammonium chloride were simultaneously administered to cirrhotic rats, the rise in blood ammonia was effectively suppressed.

**Figure 3.**
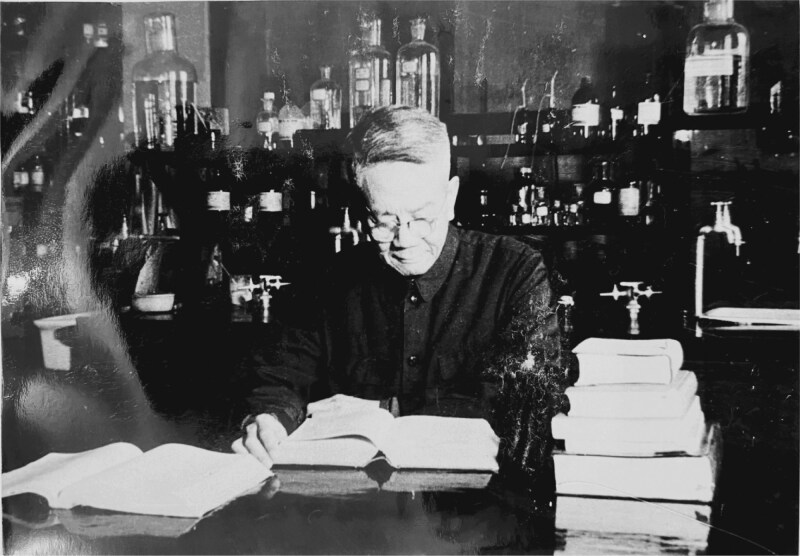
Professor Szu-Chih Liu at work (Courtesy of Peking University Health Science Center Archive).

Szu-Chih Liu and his student Tanjun Tong (童坦君) expanded their analysis to rats with liver tumor (Tong and Liu, 1965a, 1965b; Zhen and Li, 2024) (Fig. 3). In rats bearing hepatoma, blood ammonia levels were higher, and ammonia tolerance was significantly reduced, compared to normal rats. It was found that arginine, ornithine, and citrulline were capable of relieving ammonia toxicity and boost ammonia tolerance by promoting urea synthesis in rats.

## Other research interests

In 1932, Liu and Wu devised an innovative approach with an improved apparatus for measuring the electrical resistance of human skin, in which a 3-mm-thick folded layer of forearm skin was lifted and positioned between the electrodes (Liu and Wu, 1932b).

**Figure 4.**
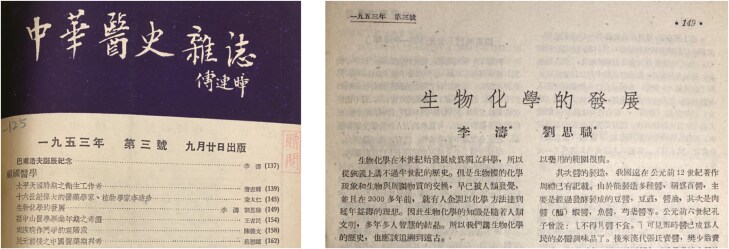
Professor Szu-Chih Liu’s research in the history of biochemistry in collaboration with the historian of medicine T’ao Lee (Courtesy of Peking University Library).

In the early 1950s, the famous historian of medicine T’ao Lee and Liu coauthored a detailed summary of biochemistry knowledge in ancient China (Lee and Liu, 1953, 1954). They asserted although biochemistry developed into an independent discipline at the dawn of the 20th century, biochemical knowledge had evolved over thousands of years alongside human civilization. It was found that, as early as the 23rd to 12th centuries BC, people in China had already observed enzymatic reactions in microbes, which were utilized in food production and disease treatment. They further discussed the enrichment of biochemistry understanding in consecutive dynasties in Chinese history (Fig. 4).

Liu formulated a comprehensive introduction of enzymology to Chinese colleagues, involving enzyme study history, enzyme properties, enzyme specificity, enzyme nomenclature and classification, coenzymes, enzyme production and application, dynamics, and kinetics of enzymatic reactions (Liu, 1955a, 1955b).

In the mid-1950s, Liu demonstrated profound enthusiasm in philosophy, discussing the origin of life, and biochemical metabolism from the perspective of dialectical materialism (Liu, 1956, 1957; Опарин et al., 1957). He also provided insightful comments on medical education in China (Cui et al., 1981). Liu crafted commemorative articles with deep affection for Professor Hsien Wu, one of the founders of modern biochemistry in China (Liu et al., 1979, 1981).

## “My ultimate aspiration was to witness biochemistry take root in China and flourish.”

Beyond his distinguished half-century academic career, Professor Szu-Chih Liu also demonstrated a profound patriotic commitment. In 1949, he declined the airplane tickets that were offered to him, choosing instead to remain resolutely in China. He once said, “I firmly believed that my career lay in my homeland. My ultimate aspiration was to witness biochemistry take root in China and flourish. I was willing to dedicate all my efforts to this cause.”(Liu, 1982). As we reflect on his contributions spanning diverse fields, Professor Liu’s work stood as a monument to the enduring power of intellectual innovation in advancing biochemistry knowledge.
